# Cytotoxic effects of different detergent containing children's toothpastes on human gingival epithelial cells

**DOI:** 10.1186/s12903-022-02089-2

**Published:** 2022-03-09

**Authors:** Sinem Birant, Yazgul Duran, Tunc Akkoc, Figen Seymen

**Affiliations:** 1 Department of Pediatric Dentistry, Faculty of Dentistry, Istanbul University-Cerrahpaşa, Istanbul, Turkey; 2 Department of Pediatric Allergy-Immunology, Faculty of Medicine, Marmara University, Istanbul, Turkey; 3 Department of Pediatric Dentistry, Faculty of Dentistry, Altinbas University, Istanbul, Turkey

**Keywords:** Toothpaste, Stem cell, Annexin V, Detergent, SLS

## Abstract

**Background:**

This study aimed to evaluate possible cytotoxic effects to gingival epithelial cells exposed to children toothpastes containing different detergent.

**Methods:**

Tissues required for the isolation of human gingival epithelial cells were obtained by biopsy during the extraction of the impacted third molar tooth. Toothpaste solutions of different concentrations were prepared from five different children’s toothpastes with different detergent contents. Isolated gingival epithelial cells were stimulated with experimental groups consisting of toothpaste solutions (Colgate, Sensodyne, Splat, Nenedent, Perlodent) at different concentrations and a control group consisting of complete Dulbecco’s modified eagle medium. After the experiments, cell viability was evaluated using flow cytometry. 2 Way ANOVA was used to see the interaction effect of the main effects of toothpaste solution and concentration factors. Pairwise comparisons were made by Tukey post hoc tests. In the study, the significance level was taken as 0.05.

**Results:**

As a result of the analysis, it was seen that the toothpaste solution and concentration factors and the interactions of these 2 factors were effective on the viable, early apoptotic, late apoptotic and necrotic cell rates. The statistically highest live cell ratios were detected in Splat’s toothpaste solutions (90.14% at 0.4% concentration) after the control group (90.82%) and the group with the lowest viability values was determined in Colgate group (75.74% at 0.4% concentration) (*p* < 0.05).

**Conclusions:**

According to the results of the study, it was observed that toothpastes containing SLS affected the viability of cells more negatively than toothpastes with other detergent contents.

## Introduction

Dental plaque is defined as a dynamic biofilm ecosystem consisting of more than 100 bacterial species, desquamated epithelial cells, salivary glycoproteins, leukocytes, macrophages and food residues that accumulate on tooth surfaces [1, 2].

Tooth decay and periodontal diseases are among the most common bacterial infections. It is reported that the most important factor of these diseases is dental plaque deposited on the tooth surface [3]. For this reason, it is critical to remove dental plaque from the tooth surface and provide oral hygiene in the prevention of tooth decay and gingival diseases. The simplest method to apply in this regard is to give individuals the habit of brushing their teeth. The most commonly used toothpastes during tooth brushing are among the most effective cosmetic and therapeutic agents in routine use, and among all dental products, they are among the most widely used by consumers [4–7].

There are many components in toothpaste, whose activities and functions are different from each other. Among these components, abrasives, water and moisturizers are present in toothpastes by 20–40%, detergents 1–2%, binding agents and sweeteners 2%, therapeutic agents 5%, colorants and preservatives 1%. The presence of these components or their concentration in toothpaste can cause undesirable side effects (such as dry mouth, recurrent aphthous and ulcers) [8, 9].

Detergents are substances that reduce surface tension known as surfactants. They have two groups, hydrophilic and hydrophobic [10, 11]. While the long hydrocarbon chain forms the water-repellent (hydrophobic) part of the molecule, it also provides the molecule with surface active properties. The polar group forms the water-loving (hydrophilic) part of the molecule and enables it to dissolve in water. The combination of these polar and apolar groups is defined as the amphiphilic structure. Thanks to the amphiphilic structure, surfactants can be dissolved in both polar and apolar solvents. While detergents adhere to water molecules with their polar parts due to these chemical properties, they ensure the removal of dirt from the environment by holding on to the dirt with their apolar parts [10–13].

Detergents are classified as anionic, cationic, amphoteric and nonionic detergents according to the ionic charge of the hydrophilic group they contain. Anionic and amphoteric detergents are frequently used in toothpaste. Sodium lauryl sulfate (SLS), sodium methy cocoyl taurate (addinol), sodium streate (sodium octadecanoate), sodium lauryl sarcosinate, sodium C12-14 olefin sulfonate, sodium C14-16 olefin sulfonate from anionic detergents and cocamidopropyl betaine (CABP) among amphoteric detergents are surfactants used frequently in toothpastes. In addition to their foaming and cleansing properties, they are routinely added to toothpastes due to their antibacterial and plaque inhibition properties [14–17].

SLS is a detergent that is often used in toothpastes with a ratio of 0.5% to 2%. SLS prevents the growth of some microorganisms by adsorption to the cell wall, penetration through the cell wall, interaction with the cell membrane, lipids and proteins, leakage of intracellular components with an increase in cell permeability and lysis in the cell [18, 19]. It has been reported in studies that SLS increases plaque inhibition, decreases *Streptococcus mutans* penetration, decreases lactate production, glucosyltransferase activity and the amount of extracellular polysaccharide created by *S. mutans* [20–22]. Despite these positive features, some toxic effects of SLS have also been reported. Oral epithelial destruction, ulcerations and inflammations caused by SLS have been observed in clinical studies. It has been reported that SLS in the toothpaste denatures the glycoproteins of the mucin layer, causing the barrier function of the oral mucosa to deteriorate, and the gingiva and buccal mucosa to be more sensitive to irritants such as exogenous antigens [23, 24]. It has also been stated that SLS may be responsible for a decrease in the keratinization level of the human oral epithelium. Sodium lauryl sulfate has also been reported to cause irritation of the oral mucosa in patients with dry mouth and the use of SLS is also associated with recurrent aphthous ulcers [23–26]. Although SLS is the most commonly used surfactant among toothpastes, surfactants with less side effects such as betaines are also used in toothpastes. Cocamidopropyl betaine, an amphoteric detergent, has been reported to have less mucosal irritation and foaming effect than SLS, and it is more biocompatible [27, 28].

There are different evaluation methods in studies conducted to determine the toxic effects of materials on cells or to investigate their biocompatibility. These tests are classified as clinical use tests, in vivo animal experiments, and in vitro cell culture tests. Cell culture tests are frequently used in cytotoxicity studies due to their ability to mimic the physiological states of living tissues. In addition, cell culture studies have many advantages such as rapid application, repetition, standardization, low cost, easy control of the experimental environment during the experiment and not being affected by different individual factors [29]. In this study, in vitro cell culture tests were preferred to determine the effects of toothpastes on cells. For this reason, in this study, it was preferred to create a primary cell culture instead of cell lines, considering the creation of experimental conditions closer to in vivo conditions. In addition, unlike other studies, not only cell viability but also apoptosis and necrosis rates were included in the study. The aim of this study was to investigate the effects of different detergent-containing children's toothpastes on the viability of human primary gingival epithelial cells.

## Materials-methods

The study was approved by the ethics committee of Istanbul University, Faculty of Dentistry (170/2017) according to Helsinki Declaration guidelines.

### Isolation and charactarization of gingival epithelial cells (GECs)

5 fully impacted human third molars, which were removed from systemically healthy patients (aged 18–25 years) were used for tissue biopsy. Gingival tissues surrounding the tooth sockets were collected immediately after tooth extraction. For the isolation of gingival epithelial cells, the gingival tissue was incubated at 4 °C in 0.4% dispase overnight. The epithelium strips were then mechanically separated and trypsinized in 0.05% trypsin/0.53 mM EDTA (Gibco, Grand Island, NY, USA) at 37 °C for 10–15 min. After strong pipetting, the cell suspension was centrifuged at 700 g for 5 min and the cell pellet was resuspended in keratinocyte growth medium (Dermalife Basal Medium; Lifeline, Walkersville, MD, USA). The cells were transferred to T-25 cm^2^ flask and were placed in the incubator which provided 5% CO_2_ environment at 37 °C. The keratinocyte growth medium was changed every 2 days and the proliferation and spreading of the cells on the flask was monitored at regular intervals by inverted miscroscope (EVOS-AMG, Thermo Fisher Scientific, Waltham, MA, USA).

Cells were fixed on the slide using 95%, 70% and 50% alcohol, respectively, at room temperature. Then, the fixation process was completed by dipping the slide into distilled water. Staining was performed with hematoxylin (Sigma-Aldrich, St. Louis, MO, USA) for 8 min. After staining, it was washed with distilled water and the second staining process was started with Eosin (Sigma-Aldrich). After staining with eosin for 90 s, the cells were dehydrated with 95% alcohol and then dipped in xylol 20 times. Microscope slides were fixed using Permount (Fisher Scientific, Pittsburgh, PA, USA) and epithelial cells were analyzed by Binocular Research Microscope (Olympus BH2-RFCA) for characterization [30].

### Preparation of toothpaste solutions

The toothpastes used in this study were Colgate 6+, Sensodyne Pronamel 6+, Nenedent (4–9 aged), Perlodent Junior 6+, Splat Juicy. The different detergent contents and other properties of these toothpastes can be seen in Table 1. Toothpaste solutions of 80%, 50%, 20% and 0.4% concentrations of these toothpastes used in the study were prepared by the method in our previous study and homogenized extraction liquids were obtained from toothpastes for cell viability experiments [31].

**Table 1:** Composition of materials evaluated

Materials	Composition	Manufacturer
Colgate 6+	Sorbitol, aqua, hydrated silica, PEG-12, *Sodium Lauryl Sulfate*, cellulose gum, sodium saccharin, sodium fluoride (1450 ppm F^−^), aroma,hydroxypropyl methylcellulose, menthol, glycerin, cinnamal, eugenol, limonene, CI 77,891, CI 42,090	Colgate Palmolive Company, Belgium
Nenedent Kids (4–9 aged)	Aqua, hydrated silica, glycerin, xylitol, propylene glycol, xanthan gum, titanium dioxide, aroma, *Sodium Lauryl Sarcosinate*, disodium EDTA, sodiummonofluorophophate (500 ppm F^−^), sodium chloride	Dentinox, Berlin, Germany
Perlodent Junior 6+	Aqua, sorbitol, hydrated silica, propylene glycol, tetrapotassium pyrophosphate, xanthan gum, *Sodium C14-16 Olefin Sulfonate,* aroma, titanium dioxide, sodium fluoride (1450 ppm F^−^), sodium saccharin, phenoxyethanol, ethylhexyl glycerin	Rossmann, Germany
Sensodyne Pronamel 6+	Aqua, sorbitol, hydrated silica, glycerin, PEG-6, *Cocamidopropyl Betaine*, xanthan gum, aroma, sodium fluoride(1450 ppm F^−^),, sodium saccharin, sucralose, titanium dioxide, sodium hydroxide, limonene	Glaxo Smith Kline, ABD
Splat Juicy	Aqua*, dicalcium phosphate dihydrate*,hydrogenated starch hydrolsate*, glycerin*, hydroxyapatite, cellulose gum*, aroma, xanthan gum*, potassium thiocyanate, lactoferrin*, lactoperoxidase*, glucose oxidase*, glucose pentaacetate, aloe barbadensis leaf extract*, sodium mthylparaben, hydrolyzed casein*, glycyrrhiza glabra root extract* (*natural origins)	SIA Splat Trading, Okulovka, Russia
Complete DMEM (CDMEM)	10% FBS (Fetal bovine serum), DMEM (Dulbecco’s Modified Eagles Medium) supplemented with 1% penicillin/streptomycin	Gibco, Grand Island, USA

### Evaluation of cell viability by flow cytometry

Gingival epithelial cells (5 × 10^5^ cells) were plated into 48-well plates separately to perform viability experiments in each concentration of toothpaste solution. The viability experiments were carried out following the method used in our previous study [31]. Gingival epithelial cells were exposed to toothpaste solutions for 2 min, washed with DPBS (Dulbecco’s phosphate buffered saline) (Gibco, Grand Island, NY, USA) and suspended in serum-free medium. 4 µL of Annexin V (BD Biosciences, CA, USA) was added to the tubes and the tubes were kept in a dark environment for 10 min. 200 µL of binding buffer was added and centrifuged at 1500 rpm for 5 min. Tubes were vortexed by adding 200 µL binding buffer. Then, 10 µL propidium iodide was added to the tubes to read the rates of viable, necrosis, early and late apoptotic cells in cells exposed to toothpaste solutions. The experiments with flow cytometry were repeated 5 times, and the average of the results obtained was calculated to determine the rates of viable, early apoptotic, late apoptotic and necrotic cells [31].

### Statistical analysis

The experiments were repeated 5 times. The average of the test results obtained was taken. The obtained datas were analyzed using the IBM SPSS V23 statistical program. 2 Way ANOVA was used to see the interaction effect of the main effects of toothpaste solution and concentration factors. Pairwise comparisons were made by Tukey post hoc tests. In the study, the significance level was taken as 0.05.

## Results

### Isolation and characterization of cells

**Fig. 1:**
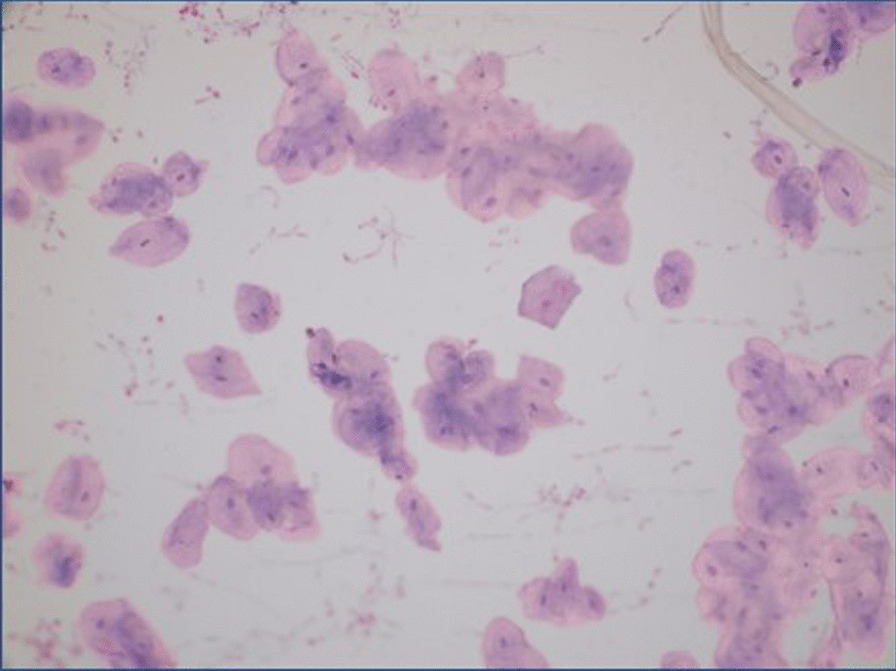
Light microscope images of GECs

It was observed that the isolated gingival epithelial cells had a cylindrical and cubic morphology by following their proliferation and reached a confluent structure from the 0th to the 3rd passage. The microscope image obtained as a result of staining with hematoxylin and eosin for the characterization of isolated gingival epithelial cells showed that the cells exhibited a cubic morphology (Fig. 1).

### Cell viability in cells cultured exposed to the children’s toothpaste containing the different detergent content

**Fig. 2:**
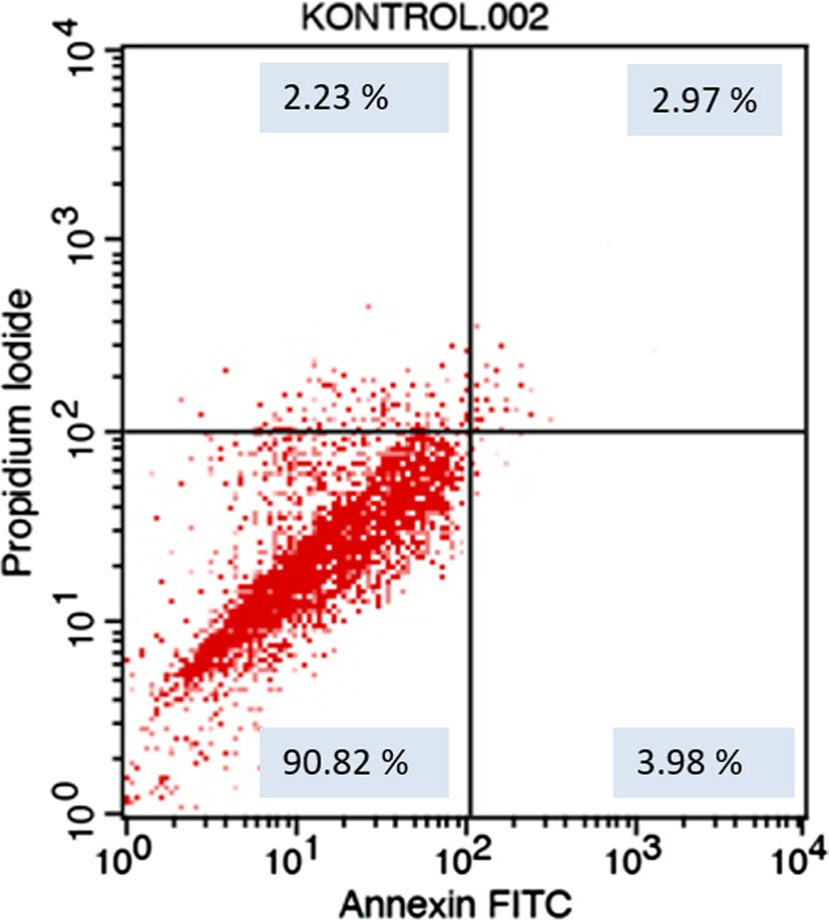
Flow cytometry graph related to the effect of CDMEM control group on gingival epithelial cells (x: Annexin V FITC, y: PIPE)

**Fig. 3:**
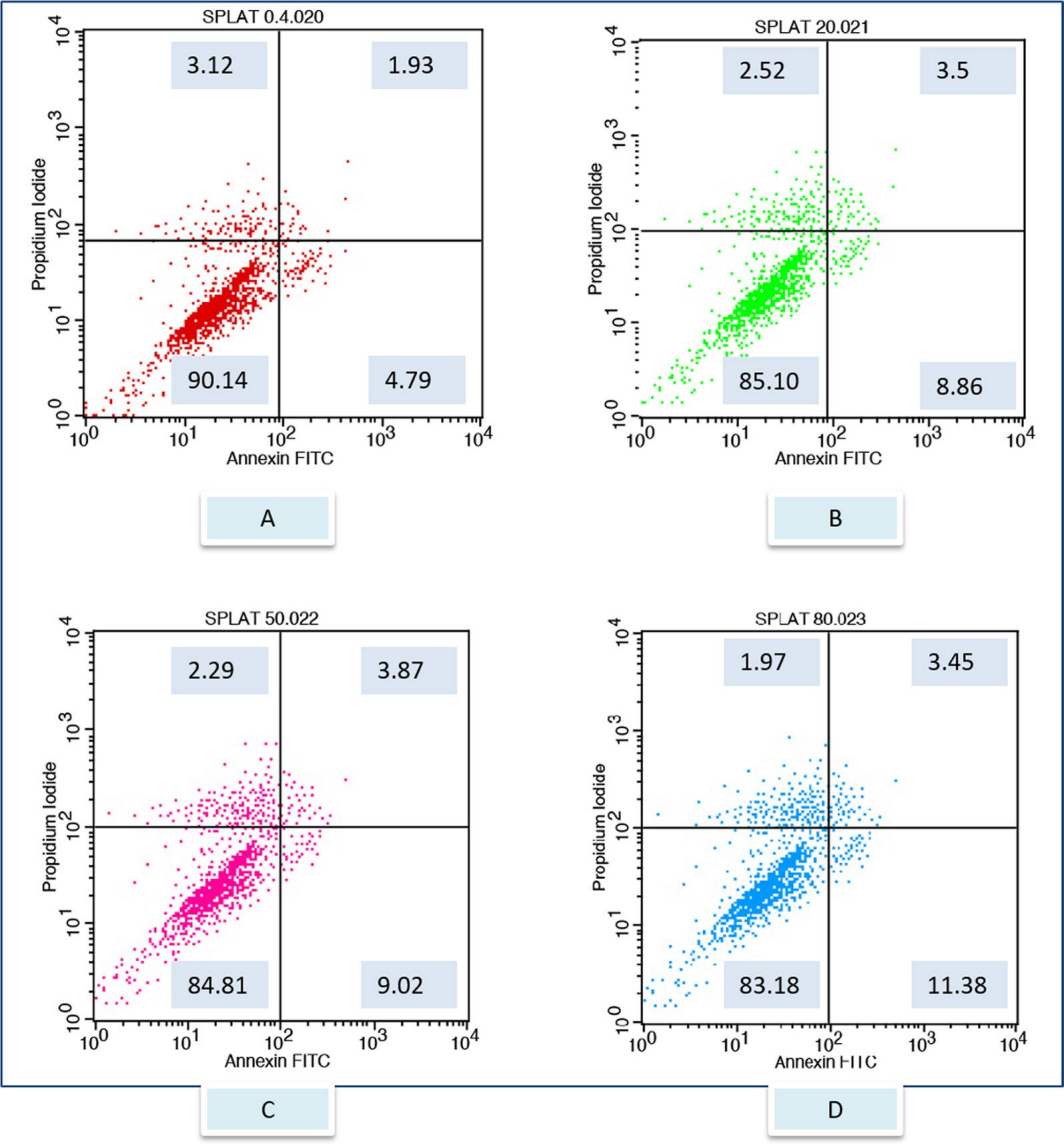
Flow cytometry graph related to the effect of Splat toothpaste solutions on gingival epithelial cells (x: Annexin V FITC, y: PIPE). **A** Splat 0.4%, **B** Splat 20%, **C** Splat 50%, **D** Splat 80%

**Fig. 4:**
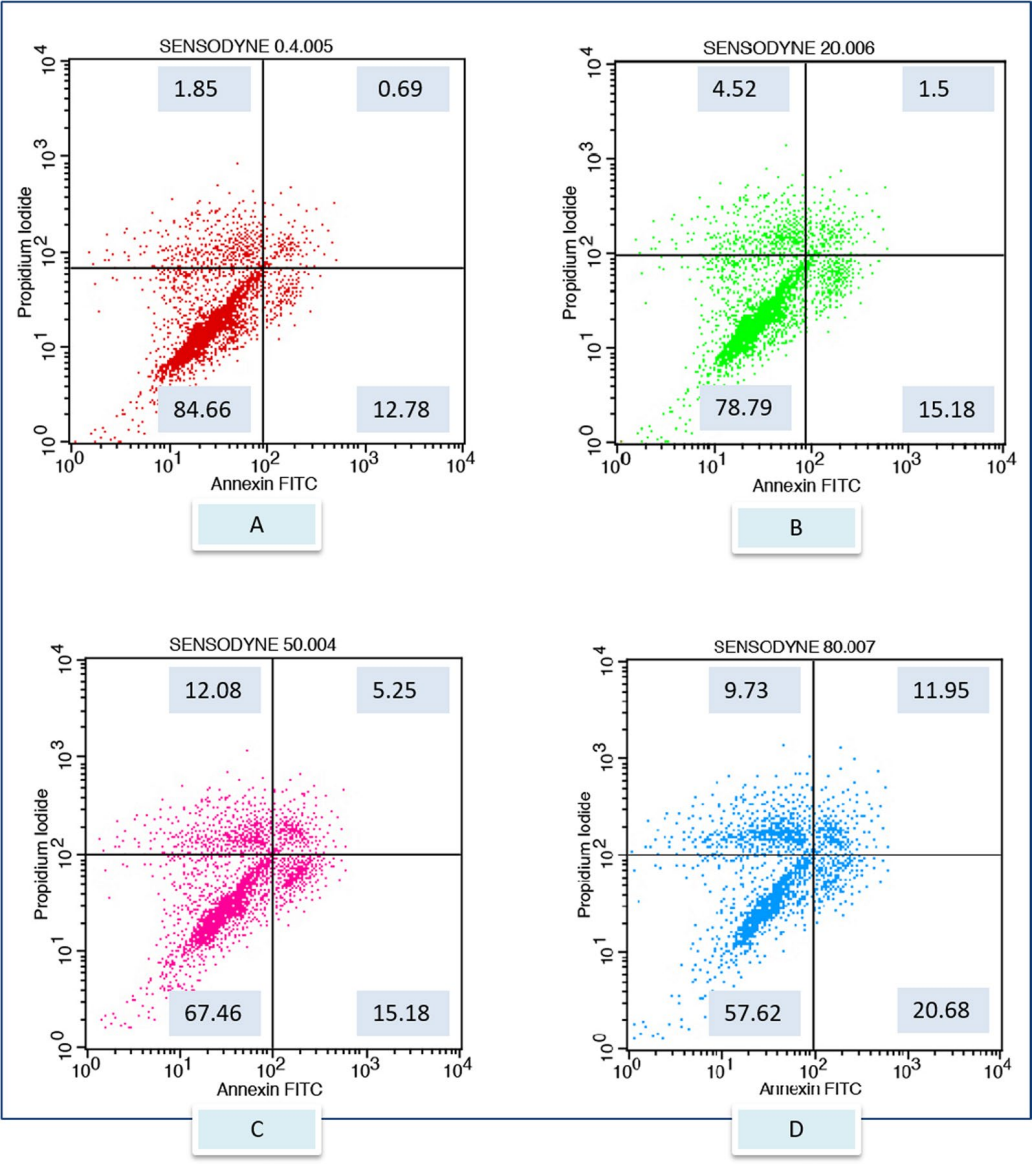
Flow cytometry graph related to the effect of Sensodyne toothpaste solutions on gingival epithelial cells (x: Annexin V FITC, y: PIPE). **A** Sensodyne 0.4%, **B** Sensodyne 20%, **C** Sensodyne 50%, **D** Sensodyne 80%

**Fig. 5:**
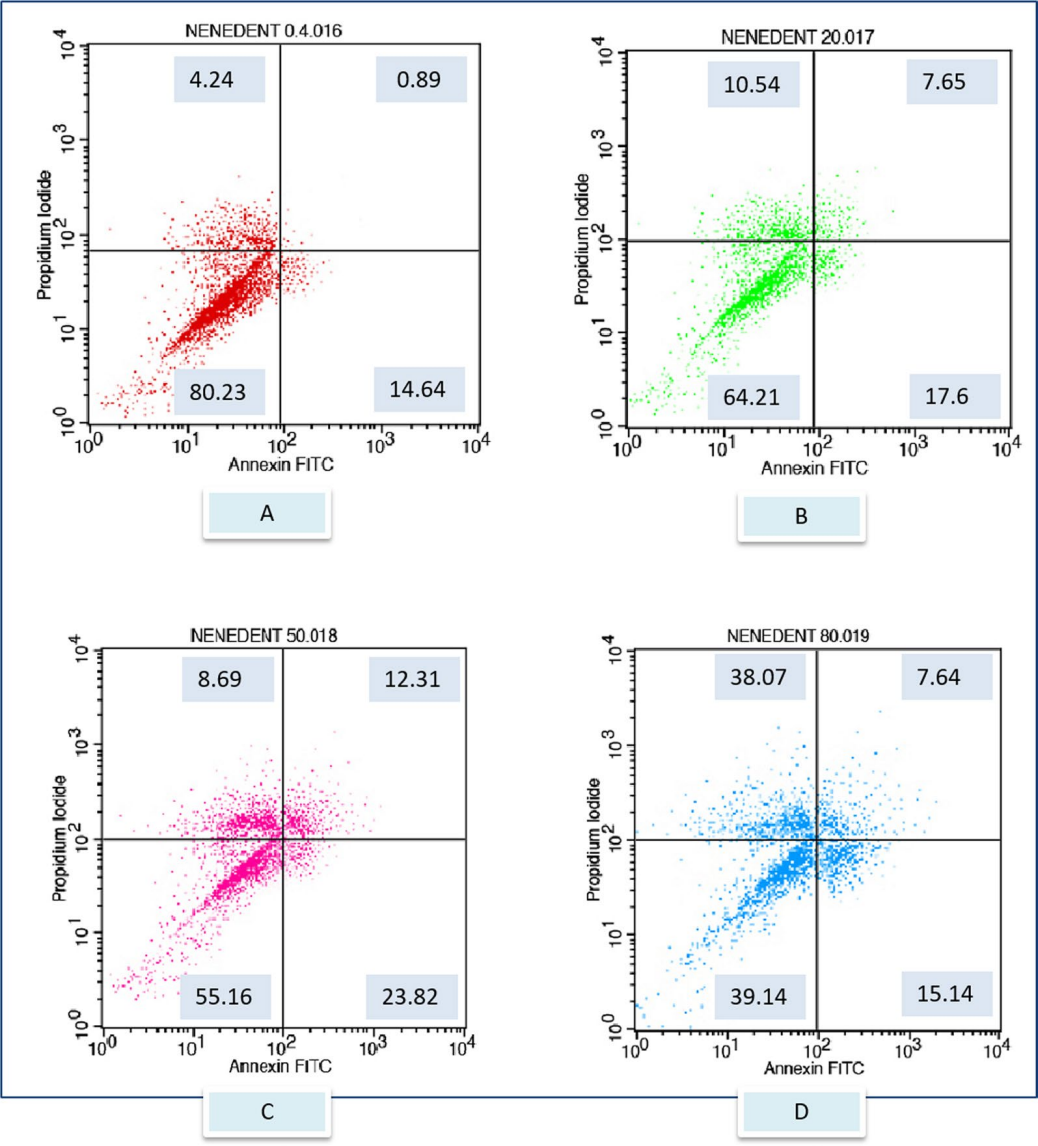
Flow cytometry graph related to the effect of Nenedent toothpaste solutions on gingival epithelial cells (x: Annexin V FITC, y: PIPE). **A** Nenedent 0.4%, **B** Nenedent 20%, **C** Nenedent 50%, **D** Nenedent 80%

**Fig. 6:**
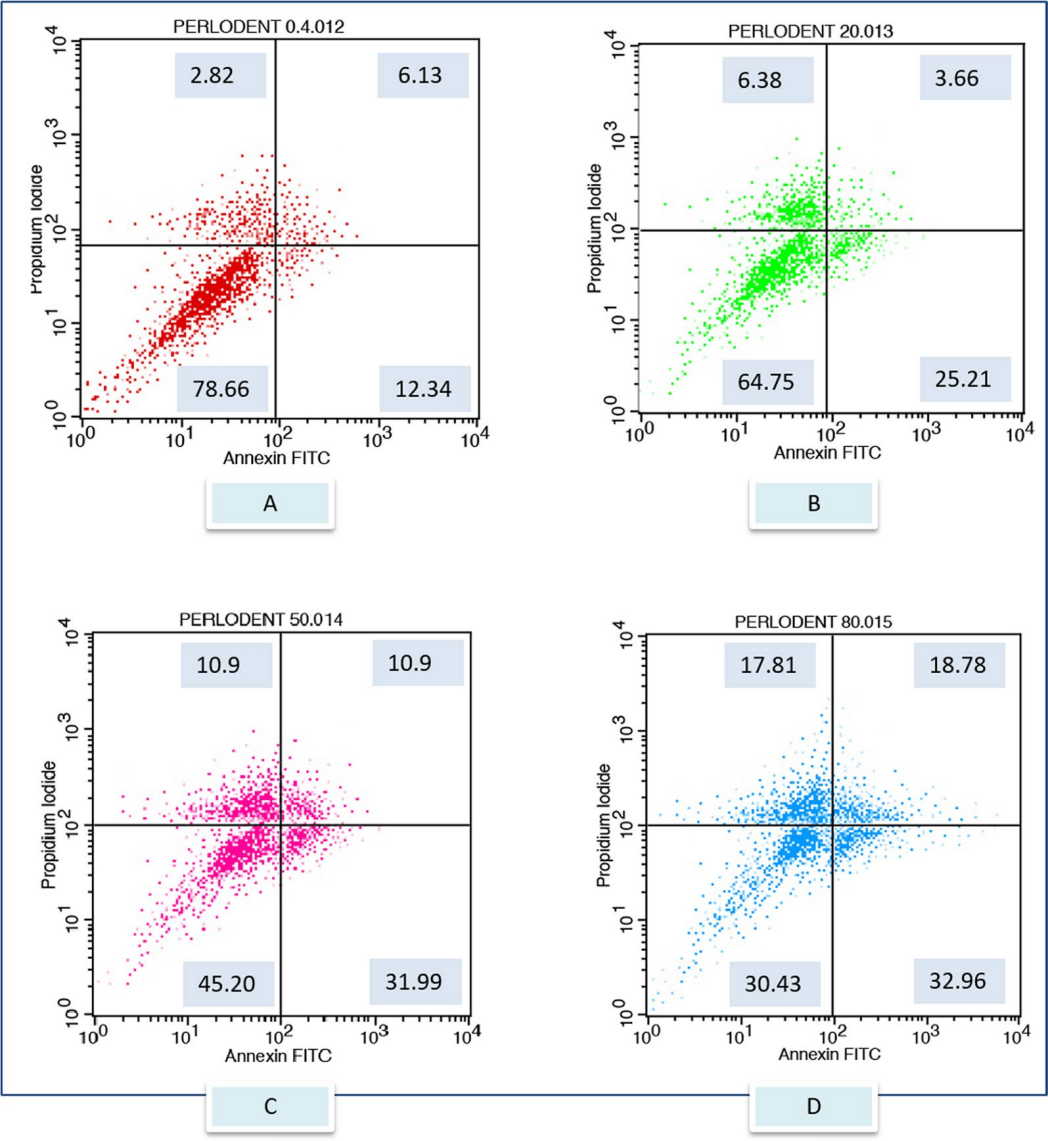
Flow cytometry graph related to the effect of Perlodent toothpaste solutions on gingival epithelial cells (x: Annexin V FITC, y: PIPE). **A** Perlodent 0.4%, **B** Perlodent 20%, **C** Perlodent 50%, **D** Perlodent 80%

**Fig. 7:**
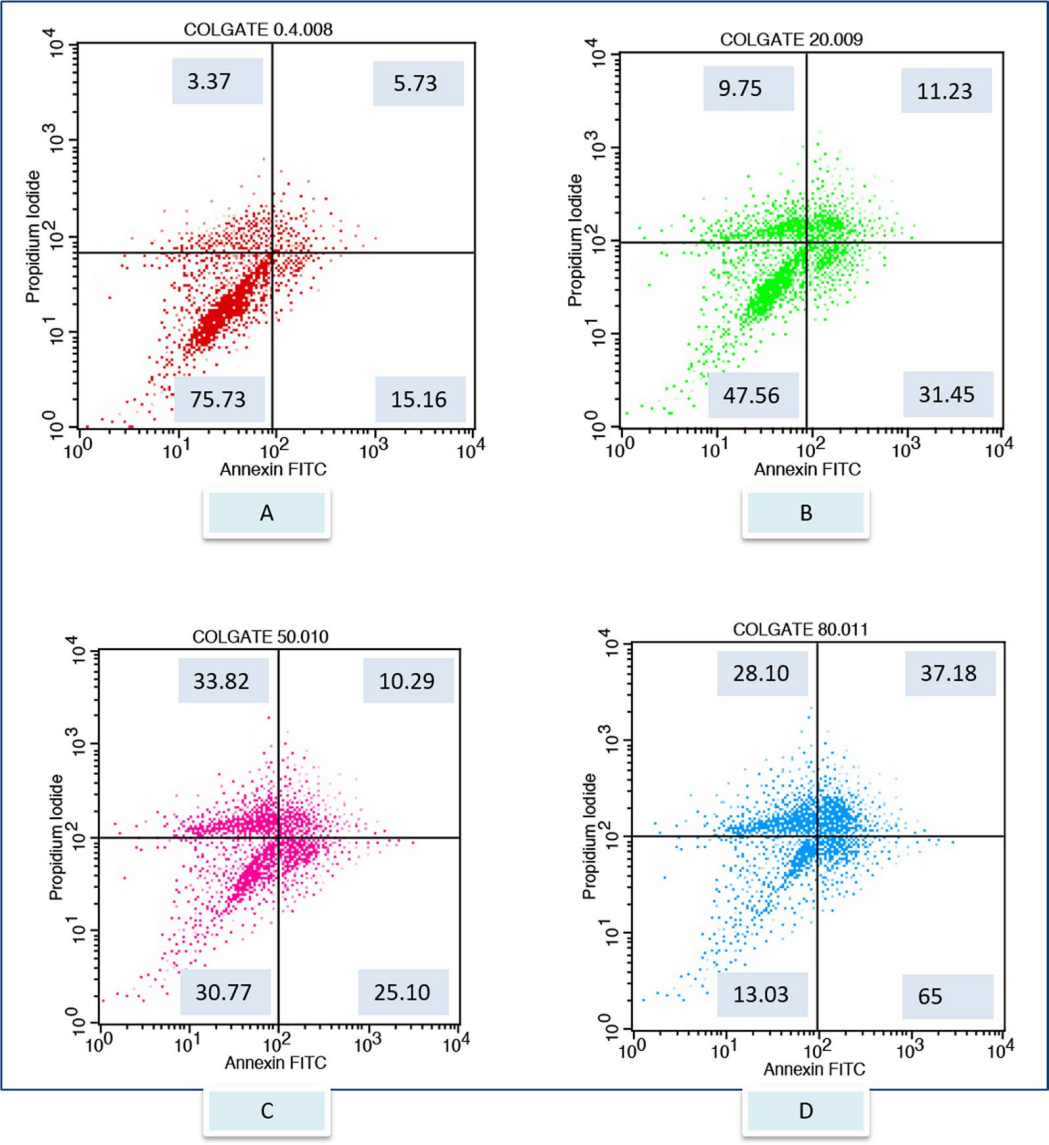
Flow cytometry graph related to the effect of Colgate toothpaste solutions on gingival epithelial cells (x: Annexin V FITC, y: PIPE). **A** Colgate 0.4%, **B** Colgate 20%, **C** Colgate 50%, **D** Colgate 80%

After exposure to the different toothpaste solutions at different toothpaste concentrations, viable and dead cell ratios were determined graphically according to Annexin-V/PI positive and negativity. Annexin V (−) and PI (−) live, Annexin V (+) and PI (−) early apoptotic cell, Annexin V (+) and PI (+) late apoptotic cell, Annexin V (−) and PI (+) considered as a necrotic cell. The flow cytometry graphs of the control group (CDMEM) in Fig. 2, the Splat group in Fig. 3, and the Sensodyne group in Fig. 4, the Nenedent group in Fig. 5, the Perlodent group in Fig. 6 and the Colgate group in Fig. 7 show the average viable, early apoptotic, late apoptotic and necrotic cell ratios.

When Table 2 is examined, it is seen that the effect of the toothpaste on cell viability is statistically significant (F = 12.781, *p* = 0.00 < 0.05). The main effect of the toothpaste on cell viability can explain 81% of the variance in viability measurements. The effect of the second factor, concentration, on cell viability is again statistically significant (F = 9.416, *p* = 0.00 < 0.05). The main effect of concentration can explain about 65% of the variance in viability measurements. In addition, the effect of the interaction of these 2 factors on cell viability was found to be statistically significant (F = 135.463, *p* = 0.00 < 0.05). The interaction effect can explain about 95% of the variance in viability measurements. In the results obtained for early apoptotic cell rates, it is seen that the effect of the toothpaste on early apoptosis is statistically significant (F = 3.063, *p* = 0.04 < 0.05). The main effect of the toothpaste could explain half of the variance in early apoptotic cell rates. The effect of the second factor, the concentration, on early apoptosis was also statistically significant (F = 3.567, *p* = 0.40 < 0.05). The main effect of concentration can explain about 42% of the variance in early apoptotic cell ratios. In addition, the effect of the interaction of these 2 factors on early apoptosis was found to be statistically significant (F = 103.589, *p* = 0.00 < 0.05). The interaction effect can explain a large part of the variance in early apoptotic cell ratios, about 94%. In the results obtained for late apoptotic cell ratios, it is seen that the effect of the toothpaste on late apoptosis is statistically significant (F = 2.966, *p* = 0.047 < 0.05). The main effect of the toothpaste could explain half of the variance in late apoptotic cell ratios. The effect of the second factor, the concentration, on late apoptosis was again statistically significant (F = 3.740, *p* = 0.04 < 0.05). The main effect of concentration can explain about 65% of the variance in late apoptotic cell ratios. In addition, the effect of the interaction of these 2 factors on late apoptosis was found to be statistically significant (F = 65.969, *p* = 0.00 < 0.05). The interaction effect can explain a large part of the variance in late apoptotic cell ratios, about 91%. In the results obtained for nectoric cell ratios, it is seen that the effect of the toothpaste on necrosis is statistically significant (F = 14.286, *p* = 0.00 < 0.05). The main effect of the toothpaste on necrosis can explain 83% of the variance in necrotic cell ratios. The effect of the second factor, the concentration, on necrosis was statistically significant again (F = 3.819, *p* = 0.03 < 0.05). The main effect of concentration can explain about 43% of the variance in necrotic cell ratios. In addition, the effect of the interaction of these 2 factors on necrosis was found to be statistically significant (F = 31.576, *p* = 0.00 < 0.05). The interaction effect can explain a large part of the variance in necrotic cell ratios, about 83%.

**Table 2:** Analysis results of the main effects of toothpaste brand and concentration factors and interaction effect on valiable, early apoptotic, late apoptotic and necrotic cell rates of gingival epithelial cells

Variable	Source	Type III Sum of Squares	df	Mean Square	F	*p*	Partial η^2^
Viable	Intercept						
	Hypothesis	546,751.350	1	546,751.350	46.157	0.000*	0.865
	Error	85,443.269	7.213	11,845.592			
	Material						
	Hypothesis	35,711.772	5	7142.354	12.781	0.000*	0.810
	Error	8382.503	15	558.834			
	Concentration						
	Hypothesis	15,786.213	3	5262.071	9.416	0.001*	0.653
	Error	8382.503	15	558.834			
	Material * Concentration						
	Hypothesis	8382.503	15	558.834	135.463	0.000*	0.955
	Error	396.033	96	4.125			
Early Apoptotic	Intercept						
	Hypothesis	10,231.641	1	10,231.641	6.100	0.055	0.543
	Error	8597.625	5.126	1677.416^a^			
	Material						
	Hypothesis	4563.250	5	912.650	3.063	0.042*	0.505
	Error	4469.342	15	297.956^b^			
	Concentration						
	Hypothesis	3188.166	3	1062.722	3.567	0.040*	0.416
	Error	4469.342	15	297.956^b^			
	Material * Concentration						
	Hypothesis	4469.342	15	297.956	103.589	0.000*	0.942
	Error	276.128	96	2.876^c^			
Late Apoptotic	Intercept						
	Hypothesis	6488.581	1	6488.581	6.699	0.049*	0.572
	Error	4860.174	5.018	968.643^a^			
	Material						
	Hypothesis	2517.559	5	503.512	2.966	0.047*	0.497
	Error	2546.763	15	169.784^b^			
	Concentration						
	Hypothesis	1904.747	3	634.916	3.740	0.035*	0.428
	Error	2546.763	15	169.784^b^			
	Material * Concentration						
	Hypothesis	2546.763	15	169.784	65.969	0.000*	0.912
	Error	247.074	96	2.574^c^			
Necrotic	Intercept						
	Hypothesis	30,236.478	1	30,236.478	18.022	0.005*	0.738
	Error	10,730.351	6.396	1677.749^a^			
	Material						
	Hypothesis	7006.300	5	1401.260	14.286	0.000*	0.826
	Error	1471.272	15	98.085^b^			
	Concentration						
	Hypothesis	1123.721	3	374.574	3.819	0.032*	0.433
	Error	1471.272	15	98.085^b^			
	Material * Concentration						
	Hypothesis	1471.272	15	98.085	31.576	0.000*	0.831
	Error	298.202	96	3.106^c^			

2 Way ANOVA

*Significant *p*-value at 0.05 level

**Fig. 8:**
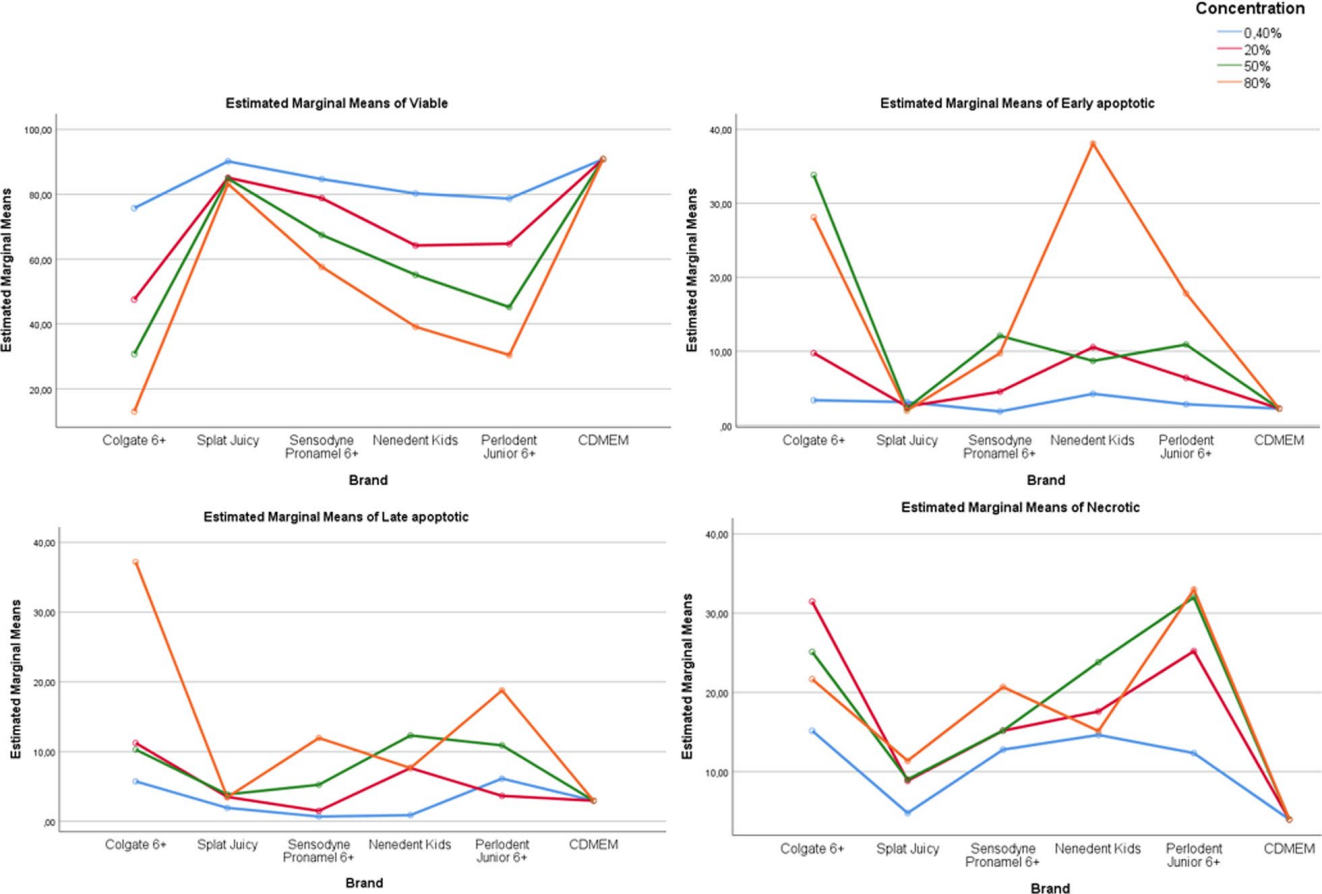
Viable, early apoptotic, late apoptotic and necrotic cell rates of concentrations for different toothpastes

When cell *viability rates* between toothpastes were compared, the difference between the means of viability at all 4 different concentration levels was statistically significant (*p* < 0.05). When comparing *early apoptotic* cell rates between toothpastes, the difference between the early apoptotic means for the 0.40% concentration was not statistically significant (*p* > 0.05). Accordingly, when the toothpastes are used with 0.40% concentration, the early apoptotic cell rates is independent of the toothpaste used. However, at the other 3 concentration levels (20%, 50%, 80%), the effect of the toothpaste on early apoptotic cell rates was statistically significant (*p* < 0.05). When *late apoptotic* cell rates between toothpastes were compared, the difference between the means of late apoptotic cell rates at all 4 different concentration levels was statistically significant (*p* < 0.05). When *necrotic* cell rates between toothpastes were compared, the difference between the means of necrotic cell at all 4 different concentration levels was statistically significant (*p* < 0.05) (Table 3, Fig. 8).

**Table 3:** Univariate tests and pairwise comparisons of viable, early apoptotic, late apoptotic and necrotic cell rates of gingival epithelial cells between toothpaste groups in each concentration levels

Variable	Brands	Concentration			
		0.40%	20%	50%	80%
Viable	Colgate 6+	75.74 ± 3.18a	47.56 ± 3.49a	30.77 ± 4.26a	13.04 ± 2.98a
	Splat Juicy	90.14 ± 0.95d	85.1 ± 1.77b	84.82 ± 1.6b	83.19 ± 1.88b
	Sensodyne Pronamel 6+	84.66 ± 1.58c	78.8 ± 1.16c	67.47 ± 1.68c	57.63 ± 0.83c
	Nenedent Kids	80.23 ± 0.93b	64.21 ± 0.91d	55.17 ± 1.2d	39.15 ± 0.91d
	Perlodent Junior 6+	78.66 ± 1.84ab	64.75 ± 0.91d	45.21 ± 1.81e	30.43 ± 4.05e
	CDMEM	90.82 ± 1.04d	9.,82 ± 1.04e	90.82 ± 1.04f	90.82 ± 1.04f
	*p*	0.000*	0.000*	0.000*	0.000*
Early apoptotic	Colgate 6+	3.38 ± 2.01	9.75 ± 3.55 cd	33.83 ± 2.81d	28.11 ± 3.07b
	Splat Juicy	3.12 ± 1.48	2.52 ± 1.14a	2.29 ± 0.78a	1.97 ± 0.4a
	Sensodyne Pronamel 6+	1.85 ± 0.53	4.52 ± 0.67ab	12.09 ± 1.11c	9.73 ± 0.57c
	Nenedent Kids	4.24 ± 0.98	10.55 ± 1.79d	8.69 ± 1.36b	38.08 ± 1.27d
	Perlodent Junior 6+	2.83 ± 0.67	6.39 ± 1.14bc	10.9 ± 0.99bc	17.82 ± 3.44e
	CDMEM	2.24 ± 1.26	2.24 ± 1.26a	2.24 ± 1.26a	2.24 ± 1.26a
	*p*	0.290	0.000*	0.000*	0.000*
Late apoptotic	Colgate 6+	5.73 ± 1.66c	11.23 ± 3.2c	10.29 ± 2.55b	37.19 ± 2.68e
	Splat Juicy	1.93 ± 0.32ab	3.51 ± 0.65a	3.87 ± 0.96a	3.46 ± 0.97ab
	Sensodyne Pronamel 6+	0.70 ± 0.37a	1.5 ± 0.37a	5.25 ± 0.64a	11.96 ± 1.02c
	Nenedent Kids	0.89 ± 0.26a	7.65 ± 1b	12.31 ± 0.53b	7.65 ± 0.6bc
	Perlodent Junior 6+	6.13 ± 1.01c	3.66 ± 0.64a	10.9 ± 1.19b	18.78 ± 4.84d
	CDMEM	2.97 ± 0.86b	2.97 ± 0.86a	2.97 ± 0.86a	2.97 ± 0.86a
	*p*	0.000*	0.000*	0.000*	0.000*
Necrotic	Colgate 6+	15.16 ± 2.4c	31.46 ± 4e	25.11 ± 2.91d	21.67 ± 2.84c
	Splat Juicy	4.79 ± 0.54a	8.86 ± 0.87b	9.03 ± 1.03b	11.39 ± 1.53b
	Sensodyne Pronamel 6+	12.79 ± 0.96bc	15.18 ± 1.09c	15.19 ± 1.28c	20.69 ± 0.91c
	Nenedent Kids	14.65 ± 0.61bc	17.6 ± 1.1c	23.83 ± 0.7d	15.15 ± 0.92b
	Perlodent Junior 6+	12.35 ± 0.68b	25.21 ± 1.3d	31.99 ± 1.2e	32.97 ± 3.99d
	CDMEM	3.98 ± 1.09a	3.98 ± 1.09a	3.98 ± 1.09a	3.98 ± 1.09a
	*p*	0.000*	0.000*	0.000*	0.000*

Each F tests the simple effects of material within each level combination of the other effects shown. These tests are based on the linearly independent pairwise comparisons among the estimated marginal means

a-b-c-d-e-f: There is no difference between the groups with the same

*Univariate Test; Tukey post-hoc Test*

*Significant *p* value at 0.05 level

**Fig. 9:**
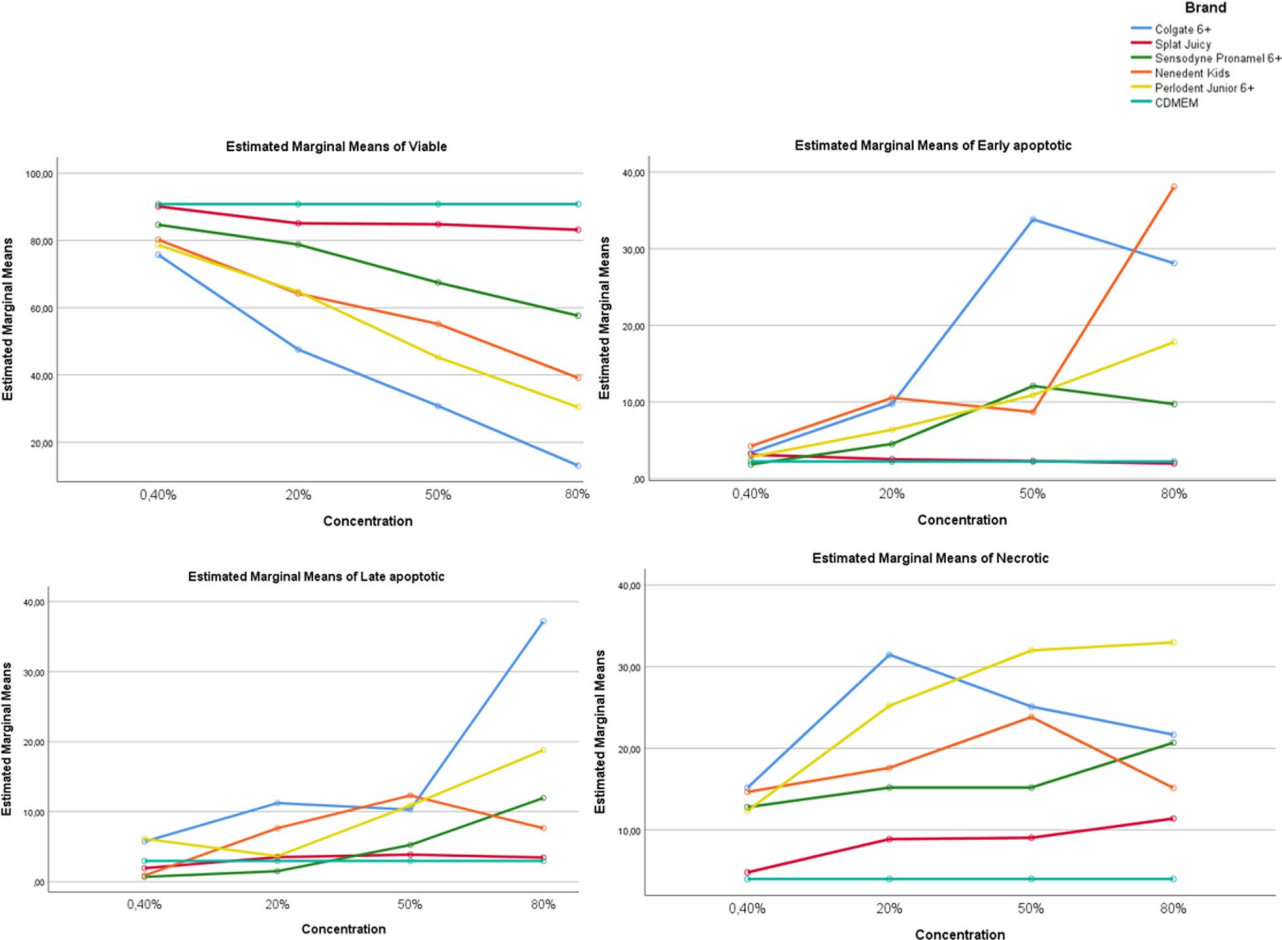
Viable, early apoptotic, late apoptotic and necrotic cell rates of toothpastes for different concentrations

When the viable, early apoptotic, late apoptotic, necrotic cell rates of the Colgate group were compared between 4 different concentration levels, at least 1 of the differences between the means was statistically significant (*p* < 0.05). Accordingly, when Colgate is used, all the measured variables are dependent on the concentration level. When the viable and necrotic cell rates of the Splat Juicy group were compared between 4 different concentration levels, at least 1 of the differences between the means was statistically significant (*p* < 0.05). Accordingly, these variables are dependent on the concentration level when Splat Juicy is used. However, on the other hand, when this material was applied with different concentrations, no significant difference was found in early and late apoptotic cell rates (*p* > 0.05). That is, apoptosis for Splat Juicy group are independent of the concentration. When the viable, early apoptotic, late apoptotic, necrotic cell rates of the Sensodyne group were compared between 4 different concentration levels, at least 1 of the differences between the means was statistically significant (*p* < 0.05). Accordingly, all the variables measured when Sensodyne is used are a variable dependent on the concentration level. When the viable, early apoptotic, late apoptotic, necrotic cell rates of Nenedent group were compared between 4 different concentration levels, at least 1 of the differences between the means was statistically significant (*p* < 0.05). Accordingly, when Nenedent is used, all of the measured variables are dependent on the concentration level. When the viable, early apoptotic, late apoptotic, necrotic cell rates of Perlodent group were compared between 4 different concentration levels, at least 1 of the differences between the means was statistically significant (*p* < 0.05). Accordingly, when Perlodent is used, all of the measured variables are dependent on the concentration level (Table 4, Fig. 9).

**Table 4:** Univariate tests and pairwise comparisons of viable, early apoptotic, late apoptotic and necrotic cell rates of gingival epithelial cells between concentration groups in each toothpaste groups

Variable	Brands	Concentration				*p*
		0.40%	20%	50%	80%	
Viable	Colgate 6+	75.74 ± 3.18A	47.56 ± 3.49B	30.77 ± 4.26C	13.04 ± 2.98D	0.000*
	Splat Juicy	90.14 ± 0.95A	85.1 ± 1.77B	84.82 ± 1.6B	83.19 ± 1.88B	0.000*
	Sensodyne Pronamel 6+	84.66 ± 1.58A	78.8 ± 1.16B	67.47 ± 1.68C	57.63 ± 0.83D	0.000*
	Nenedent Kids	80.23 ± 0.93A	64.21 ± 0.91B	55.17 ± 1.2C	39.15 ± 0.91D	0.000*
	Perlodent Junior 6+	78.66 ± 1.84A	64.75 ± 0.91B	45.21 ± 1.81C	30.43 ± 4.05D	0.000*
Early apoptotic	Colgate 6+	3.38 ± 2.01A	9.75 ± 3.55B	33.83 ± 2.81C	28.11 ± 3.07D	0.000*
	Splat Juicy	3.12 ± 1.48	2.52 ± 1.14	2.29 ± 0.78	1.97 ± 0.4	0.745
	Sensodyne Pronamel 6+	1.85 ± 0.53A	4.52 ± 0.67B	12.09 ± 1.11C	9.73 ± 0.57C	0.000*
	Nenedent Kids	4.24 ± 0.98A	10.55 ± 1.79B	8.69 ± 1.36B	38.08 ± 1.27C	0.000*
	Perlodent Junior 6+	2.83 ± 0.67A	6.39 ± 1.14B	10.9 ± 0.99C	17.82 ± 3.44D	0.000*
Late apoptotic	Colgate 6+	5.73 ± 1.66A	11.23 ± 3.2B	10.29 ± 2.55AB	37.19 ± 2.68C	0.000*
	Splat Juicy	1.93 ± 0.32	3.51 ± 0.65	3.87 ± 0.96	3.46 ± 0.97	0.238
	Sensodyne Pronamel 6+	0.70 ± 0.37A	1.5 ± 0.37A	5.25 ± 0.64B	11.96 ± 1.02C	0.000*
	Nenedent Kids	0.89 ± 0.26A	7.65 ± 1B	12.31 ± 0.53C	7.65 ± 0.6B	0.000*
	Perlodent Junior 6+	6.13 ± 1.01A	3.66 ± 0.64A	10.9 ± 1.19B	18.78 ± 4.84C	0.000*
Necrotic	Colgate 6+	15.16 ± 2.4A	31.46 ± 4B	25.11 ± 2.91C	21.67 ± 2.84C	0.000*
	Splat Juicy	4.79 ± 0.54A	8.86 ± 0.87B	9.03 ± 1.03B	11.39 ± 1.53C	0.000*
	Sensodyne Pronamel 6+	12.79 ± 0.96A	15.18 ± 1.09B	15.19 ± 1.28B	20.69 ± 0.91C	0.000*
	Nenedent Kids	14.65 ± 0.61A	17.6 ± 1.1B	23.83 ± 0.7C	15.15 ± 0.92A	0.000*
	Perlodent Junior 6+	12.35 ± 0.68A	25.21 ± 1.3B	31.99 ± 1.2C	32.97 ± 3.99C	0.000*

Each F tests the simple effects of concentration within each level combination of the other effects shown. These tests are based on the linearly independent pairwise comparisons among the estimated marginal means

A-B-C-D: There is no difference between the groups with the same

*Univariate Test,; Tukey post-hoc Test*

*Significant *p* value at 0.05 level

## Discussion

Detergents, one of the toothpaste components, are frequently used in removing plaque, due to their antimicrobial properties. However, it is stated that besides these positive properties, they also have the potential to adversely affect the oral mucosa [32]. In this study, when the viability rates of different detergent-containing children's toothpaste solutions on cells were evaluated, it was observed that the lowest viable cell rates were in SLS-containing toothpaste solutions. After the control group, the highest vitality values were determined in the toothpaste without detergent content, followed by the toothpaste containing CAPB.

Clinical intraoral side effects such as mucosal sensitivity, epithelial desquamation and recurrent aphthous ulcerations in vitro studies point to the possible problems of these ingredients used in adult toothpaste [27, 33, 34]. Studies examining the effects of these components in children's toothpastes on intraoral tissues are very few. When looking at the contents of children's toothpaste, it is seen that many paste contents contain SLS as a type of detergent. However, considering the side effects of SLS, the different degree of keratinization and morphology of the gingival of children suggests that these side effects may occur more in children.

Therefore, in this study, the effects of 5 different children's toothpaste with different detergent content on cells were investigated. There are different evaluation methods to investigate the effects on cells, to determine the toxic effects of the materials to be used or to investigate their biocompatibility. These tests can be classified as clinical use tests, in vivo animal experiments and in vitro cell culture tests. Among these alternative methods, cell culture tests are frequently used in cytotoxicity studies due to their ability to mimic the physiological conditions of living tissues. In addition, cell culture studies have many advantages such as rapid application, repeatability, standardization, low cost, easy control of the experimental environment during the experiment and not being affected by different individual factors [35, 36]. Since there are some ethical and legal problems in other test methods, in vitro cell culture tests constitute the starting point of such studies in biocompatibility and cytotoxicity studies. In this study, in vitro cell culture tests were preferred to determine the effects of toothpastes on cells.

The cell type used in cell culture studies should be selected in relation to the area of use of the material whose cytotoxic effects are investigated. Primary cell cultures or continuous cell lines are used in studies as a biological system in biocompatibility tests. It is stated that continuous cell lines such as L929, 3T3, HSC-2, MRC-5 can be used in the cytotoxicity assessment tests of materials used in dentistry, since they can be obtained more easily than primary cell cultures and have rapid reproduction potential. However, since primary cell cultures are more sensitive than continuous cell lines, they are biological systems that best reflect the original physiological state, despite the difficulties that arise during the production phase and the long time to produce [30, 35, 37–41]. For this reason, it was preferred to create a primary cell culture in this study, considering the creation of experimental conditions closer to in vivo conditions. Gingival epithelial cells were used as a biological system in this study, since the majority of oral tissues that toothpastes come into contact with during tooth brushing are gingival tissues.

In studies for the characterization of gingival epithelial cells, the method of determining epithelial cells specific CK13 and Vimentin genes by PCR, analysis of phenotypic properties of cells by transmission electron microscopy, determination of a specific epithelial marker cytokeratin by immunofluorescence method, staining of cells with Papanicolau staining method and analysis under light microscopy. methods were used [42]. In this study, the cells, which are easier and quicker to apply than other methods and are also more cost-effective, were stained with hematoxylin and eosin dyes after fixation with alcohol on the slide, and the cells were analyzed under light microscopy, and the presence of epithelial cells was determined.

Many in vitro tests such as MTT, trypan blue exclusion test, micronucleus are used to determine cell viability [27, 28, 43–48]. Flow cytometry analysis is frequently recommended in terms of providing more reliable, faster and more sensitive results than other methods in evaluating cell viability and cytotoxicity [29]. In addition to determining cell viability, information about different properties of cells such as immunophenotypic properties, enzyme activities, and specific markers of the cell can be obtained with this method [49, 50]. In addition, the separation of apoptotic and necrotic cells with this method is important in terms of different biological responses of these two types of death [49, 51]. In this study, since gingival cells are labeled with Annexin V and propidium iodide dyes, since they give faster, more sensitive and reliable results compared to alternative methods used in cell culture studies, it was ensured that live, early apoptotic, late apoptotic and necrotic cells were determined by flow cytometry analysis.

In the literature, changes caused by SLS, which is frequently used in toothpaste, on the oral mucosa have been reported. In addition, in a few studies examining the effects of SLS on cells, it has been stated that they have a negative effect on cell viability [27, 28, 52–57]. In this study, SLS, sodium lauryl sarcosinate, sodium C14-16 olefin sulfonate, CAPB containing toothpastes which are reported to be more biocompatible than SLS, toothpaste without detergent and CDMEM were selected as experimental groups. While determining the concentrations of toothpaste solutions in cell viability experiments, similar studies have been examined and optimized as 0.4%, 20%, 50% and 80%. In addition, in this study, the stimulation time of toothpaste solutions with cells was determined as 2 min, since the brushing time was 2 min under normal conditions [27, 28, 43].

When the studies on detergents are examined; Herlofson et al. found a positive relationship between oral desquamation and SLS in their study [58]. Melsen et al. examined the effect of SLS on monoflurophosphate, and it was stated that SLS reduced the amount of fluoride taken up by the enamel [59]. Rantanen et al. reported that toothpastes containing SLS have an irritating effect on the mucosa [60]. Shim et al. investigated the effect of SLS on recurrent aphthous stomatitis and showed that SLS significantly increased the incidence of ulcers, the duration of ulcers in the mouth, and the pain score [56].

In this study, when the viability rates of different detergent-containing children's toothpaste solutions on human gingival epithelial cells were evaluated, it was seen that the lowest proportion of viable cells was in toothpaste solutions containing SLS. After the control group, the highest vitality values were detected in toothpaste without detergent content, followed by toothpaste containing CAPB. The effects of this study on cell viability Cvikl et al.'s findings in studies examining the effects of adult toothpastes and children's toothpaste on cells [27, 28]. Moore et al. also found that cell viability rates in SLS and betaine containing toothpastes were lower than the control group. These findings are also similar to the findings in our study.

In the literature, the effects of toothpastes on cells have been examined only in terms of living cell proportions [27, 28]. In this study, early apoptotic, late apoptotic and necrotic cell ratios were evaluated as well as the live cell ratios. In the comparisons between the groups, the Colgate group generally shows the highest value in terms of early apoptotic, late apoptotic and necrotic cell ratios, while Splat and the control group generally have similar values in terms of cell death type rates. Considering that SLS increases cellular permeability by causing denaturation of cellular proteins in this study, we think that the opening of the pores between cells may cause the release of apoptosis-inducing proteins into the cytosol and ultimately stimulate apoptosis/necrosis mechanisms. It has been reported that stimulation of apoptosis and necrosis mechanisms in gingival epithelial cells may prevent periodontal wound healing and prolong the healing period [61, 62]. In this study, it is thought that the increase in the ratio of apoptotic and necrotic cells of SLS-containing toothpaste may delay the healing time of periodontal diseases and oral aphthous ulcers and adversely affect wound healing.

This study has some limitations due to the absence of saliva, the protective and immunological properties of tissue barriers. In addition, this study suggests that other ingredients in toothpaste may also have toxic effects, since detergent ingredients cannot be supplied in pure form. However, in order to eliminate this limitation, toothpastes used in similar age groups and having similar contents formed the study groups in our study.

## Data Availability

The datasets used and/or analysed during the current study available from the corresponding author on reasonable request.

## Abbreviations

GEC: Gingival epithelial cells
SLS: Sodium lauryl sulfate
CDMEM: Complete dulbecco’s modified eagles medium
CABP: Cocamidopropyl betaine
DPBS: Dulbecco’s phosphate buffered saline

## References

[1] Rosan B, Lamont RJ (2000). Dental plaque formation. Microb Infect.

[2] Takeshita T, Yasui M, Shibata Y, Furuta M, Saeki Y, Eshima N (2015). Dental plaque development on a hydroxyapatite disk in young adults observed by using a barcoded pyrosequencing approach. Sci Rep.

[3] Walsiluk A (2017). Fluoride compounds in dental caries prophylaxis in children and adolescents—review of polish literature. Przegl Epidemiol.

[4] Claydon NC (2000). Current concepts in toothbrushing and interdental cleaning. Periodontol.

[5] Trubey RJ, Moore SC, Chestnutt IG (2014). Parents reasons for brushing or not brushing their child’s teeth: a qualitative study. Int J Paediatr Dent.

[6] Deery C, Heanue M, Deacon S, Robinson PG, Walmsley AD, Worthington H (2004). The effectiveness of manual versus powered toothbrushes for dental health: a systematic review. J Dent.

[7] Wilder RS, Bray KS (2000). Improving periodontal outcomes: merging clinical and behavioral science. Periodontol.

[8] Welbury R, Duggal M, Hosey TM (2005). Paediatric dentistry.

[9] Ozkocak CBB, Karaarslan SE, Aytac F (2017). Saliva proteins and their effects on caries. Turkey Dental Clinics J Sci.

[10] Chambers HF, Katzung BG (2007). Miscellaneous antimicrobial agents; disinfectants, antiseptic, sterilants. Basic & clinical pharmacology.

[11] Ozyurt M, Gunaydın M, Sanic A, Gurler B (2005). Aldehyde, peroxygen and peracetic acid and other disinfectants that do not contain chlorine donating agents and are recommended as instrument disinfectants, their general use and antimicrobial effectiveness. 4th national sterilization disinfection congress, congress book.

[12] Ananthapadmanabhan KP, Moore DJ, Subramanyan K, Misra M, Meyer F (2004). Cleansing without compromise: the impact of cleansers on the skin barrier and the technology of mild cleansing. Dermatol Ther.

[13] Walters KA, Bialik W, Brain KR (1993). The effects of surfactants on penetration across the skin. Int J Cosmetics Sci.

[14] Shah SK, Niraula TP, Bhattarai A, Chatterjee SK (2012). A comparative study of cationic and anionic surfactants on the micellar behavior through different composition of methanol-water mixed solvent. Conductometric Method Bibechan.

[15] Forward GC, James AH, Barnett P, Jackson RJ (2000). Gum health product formulations: what is in them and why?. Periodontol.

[16] Petersen FC, Assev S, Scheie AA (2006). Combined effects of NaF and SLS on acid and polysaccharide formation of biofilm and planktonic cells. Arch Oral Biol.

[17] Buma R, Maeda T, Kamei M, Kourai H (2006). Pathogenic bacteria carried by companion animals and their susceptibility to antibacterial agents. Biocontrol Sci.

[18] Law V, Seow WK (2006). A longitudinal controlled study of factors associated with mutans streptococci infection and caries lesion initiation in children 21 to 72 months old. Pediatr Dent.

[19] Nordstrom A, Mystikos C, Ramberg P, Birkhed D (2009). Effect on denovo plaque formation of rinsing with toothpaste slurries and water solutions with a high fluoride concentration (5,000 ppm). Eur J Oral Sci.

[20] Moran J, Addy M, Newcombe R (1988). The antibacterial effect of toothpastes on the salivary flora. J Clin Periodontol.

[21] Moran J, Addy M, Newcombe R (1989). Comparison of the effect of toothpastes containing enzymes or antimicrobial compounds with a conventional fluoride toothpaste on the development of plaque and gingivitis. J Clin Periodontol.

[22] Evans A, Leishman SJ, Walsh LJ (2015). Inhibitory effects of children’s toothpastes on *Streptococcus mutans*, *Streptococcus sanguinis* and *Lactobacillus acidophilus*. Eur Arch Paediatr Dent.

[23] Macdonald JM, Tobin CA, Burkemper NM, Hurley MY (2015). Oral Leukoedema with mucosal desquamation caused by toothpaste containing sodium lauryl sulfate. Case Let.

[24] Neppelberg E, Costea DE, Vintermyr OK, Johannessen AC (2007). Dual effects of sodium lauryl sulphate on human oral epithelial structure. Experiment Dermatol.

[25] Siegel IA, Gordon HP (1986). Surfactant-induced alterations of permeability of rabbit oral mucosa in vitro. Exp Mol Pathol.

[26] Ahlfors EE, Lyberg T (2001). Contact sensitivity reactions in the oral mucosa. Acta Odontol Scand.

[27] Cvikl B, Lussi A, Moritz A, Gruber R (2015). The in vitro impact of toothpaste extracts on cell viability. Eur Oral Sci.

[28] Cvikl B, Lussi A, Moritz A, Gruber R (2017). Dentifrices for children differentially affect cell viability in vitro. Clin Oral Invest.

[29] Zhou H, Shen Y, Wang Z, Li L, Zheng Y, Hakkinen L (2013). In vitro cytotoxicity evaluation of a novel root repair material. J Endod.

[30] Russo FB, Pignatari GC, Fernandes IR, Dias JLRM, Beltrao-Braga PCB (2016). Epithelial cells from oral mucosa: how to cultivate them?. Cytotechnol.

[31] Birant S, Duran Y, Gokalp M, Akkoc T, Seymen F (2021). Effects of different-containing children’s toothpastes on the viability, osteogenic and chondrogenic differentiation of human dental periodontal ligament stem cells and gingival stem cells in vitro. Tissue Cell.

[32] Lawrence LM, Farquharson A, Brown RS, Vatanka HO (2013). Oral tissue irritants in toothpaste: a case report. J Clin Pediatr Dent.

[33] Herlofson BB, Barkvoll P (1996). Oral mucosal desquamation caused by two toothpaste detergents in an experimental model. Eur J Oral Sci.

[34] Skaare AB, Rolla G, Barkvoll P (1997). The influence of triclosan, zinc or propylene glycol on oral mucosa exposed to sodium lauryl sulphate. Eur J Oral Sci.

[35] Craig RG, Powers JM, Sakaguchı RL (2006). Craig’s restorative dental materials.

[36] Schmalz G (1994). Use of cell cultures for toxicity testing of dental materials- advantages and limitations. J Dent.

[37] Arenholt-Bindslev D, Bleeg H (1990). Characterization of two types of human oral fibroblast with a potential application to cellular toxicity studies; tooth pulp fibroblasts and buccal mucosa fibroblasts. Int Endod J.

[38] Illeperuma RP, Park YJ, Kim JM, Bae JY, Che ZM, Son HK (2012). Immortalized gingival fibroblasts as a cytotoxicity test model for dental materials. J Mater Sci Mater Med.

[39] International Organization for Standardization. Dentistry-Biological evaluation of medical devices. Tests for in vitro cytotoxicity. ISO 10993-5. 2009. https://www.iso.org/standard/36406.html. Accessed 13 Feb 2019.

[40] Schmalz G (1997). Concepts in biocompatibility testing of dental restorative materials. Clin Oral Invest.

[41] Tuncer S, Demirci M (2011). Biocompatibility evaluations of dental materials. J Atatürk Univ Dentist.

[42] Ghapanchi J, Kamali F, Moattari A, Poorshahidi S, Shahin E, Rezazadeh F (2015). In vitro comparison of cytotoxic and antibacterial effects of 16 commercial toothpastes. J Int Oral Health.

[43] Fiori J, Teti G, Gotti R, Mazzotti G, Falconi M (2011). Cytotoxic activity of guaiazulene on gingival fibroblasts and influence of light exposure on guaiazzulene-induced cell death. Toxico In Vitro.

[44] Eyuboglu GB, Yesilyurt C, Erturk M (2015). Evaluation of cytotoxicity of dentin desensitizing products. Oper Dentist.

[45] Kalil Bussadori S, Marcilio Santos E, Cardoso Guedes C, Jansiski Motta L, Santos Fernandes KP, Mesquita-Ferrari RA (2010). Cytotoxicity assessment of casein phosphopeptide-amorphous calcium phosphate (CPP-ACP) paste. ConScient Saude.

[46] Fernandes JPS, Mello Moura ACV, Marques MM, Nicoletti MA (2012). Cytotoxicity evaluation of Curcuma zedoaria Roscoe fluid extract used in oral hygiene products. Acta Odontol Scand.

[47] Olgun Erdemir E, Sengun A, Ulker M (2007). Cytotoxicity of mouthrinses on epithelial cells by micronucleus test. Eur J Dent.

[48] Kanev MO, Gokalp Muranlı FD (2016). Flow cytometry and usage areas. J SAÜ Fen Bil.

[49] Coskun G, Ozgur H (2011). Molecular mechanism of apoptosis and necrosis. Arch Med Rev J.

[50] Salzer S, Rosema NAM, Maertin ECJ, Slot DE, Timmer CJ, Dorfer CE (2016). The effectiveness of dentifrices without and with sodium lauryl sulfate on plaque, gingivitis and gingival abrasion—a randomized clinical trial. Clin Oral Invet.

[51] Healy CM, Paterson M, Joyston-Bechal S, Williams DM, Thornhill MH (1999). The effect of a sodium lauryl sulfate-free dentifrice on patients with recurrent oral ulceration. Oral Dis.

[52] Barkvoll P, Rolla G, Svendsen AK (1989). Interaction between chlor- hexidine digluconate and sodium lauryl sulfate in vivo. J Clin Periodontol.

[53] Allen AL, Hawley CE, Cutright DE, Seibert JS (1975). An investigation of the clinical and histologic effects of selected dentifrices on human palatal mucosa. J Periodontol.

[54] Shim YJ, Choi JH, Ahn HJ, Kwon JS (2012). Effect of sodium lauryl sulfate on recurrent aphthous stomatitis: a randomized controlled clinical trial. Oral Dis.

[55] Moore C, Addy M, Moran J (2008). Toothpaste detergents: a potential source of oral soft tissue damage?. Int J Dent Hyg.

[56] Herlofson BB, Brodin P, Aars H (1996). Increased human gingival blood flow induced by sodium lauryl sulfate. J Clin Periodontol.

[57] Melsen B, Rolla G (1983). Reduced Clinical effect of monofluorophosphate in the presence of sodium lauryl sulphate. Caries Res.

[58] Rantanen I, Jutila K, Nicander I, Tenovuo J, Soderling E (2003). The effects of two sodium lauryl sulphate-containing toothpastes with and without betaine on human oral mucosa in vivo. Swed Dent J.

[59] Semlali A, Chakir J, Goulet JP, Chmielwski W, Rouabhia M (2011). Whole cigarette smoke promets human gingival epithelial cell apoptosis and inhibits cell repair processes. J Periodont Res.

[60] Rouabhia M (2002). Interactions between host and oral commensal microorganisms are key events in health and disease status. Can J Infect Dis.

[61] Weindl G, Wagener J, Schaller M (2010). Epithelial cells and innate antifungal defense. J Dent Res.

[62] Bahri R, Saidane-Mosbahi D, Rouabhia M (2010). Candida famata modulates toll-like receptor, beta-defensin, and proinflammatory cytokine expression by normal human epithelial cells. J Cell Physiol.

